# Synergistic associations of CD33 variants and hypertension with poor cognitive function, brain atrophy, and cerebral blood flow among dementia‐free older adults: a population‐based study

**DOI:** 10.1002/alz.088540

**Published:** 2025-01-03

**Authors:** Min Zhu, Xunyao Tian, Xiaodong Han, Cuicui Liu, Na Tian, Heng Zhang, Lin Song, Shi Tang, Lin Cong, Yongxiang Wang, Tingting Hou, Chengxuan Qiu, Yifeng Du

**Affiliations:** ^1^ Shandong Provincial Hospital Affiliated to Shandong First Medical University, Jinan China; ^2^ Shandong Provincial Hospital, Cheeloo College of Medicine, Shandong University, Jinan China; ^3^ Shandong Provincial Clinical Research Center for Neurological Diseases, Jinan China; ^4^ Xuanwu Hospital, Capital Medical University, Beijing, Beijing China; ^5^ Department of Neurology, Shandong Provincial Hospital, Shandong University, Jinan, Shandong, P.R. China., Jinan China; ^6^ Department of Neurology, Shandong Provincial Hospital Affiliated to Shandong First Medical University, Jinan, Shandong China; ^7^ Shandong Provincial Hospital affiliated to Shandong First Medical University, Jinan China; ^8^ Shandong Provincial Hospital, Shandong University, Jinan China; ^9^ Shandong Provincial Hospital Affiliated to Shandong University, Jinan China; ^10^ Shandong Provincial Clinical Research Center for Neurological Diseases, Jinan, Shandong, P.R. China., Jinan China; ^11^ Aging Research Center and Center for Alzheimer Research, Karolinska Institutet‐Stockholm University, Stockholm Sweden; ^12^ Shandong Provincial Hospital Affiliated to Shandong First Medical University, Jinan, Shandong China

## Abstract

**Background:**

Evidence has emerged that the inflammation and immune mechanisms are involved in the relationship between hypertension (HTN) and cognitive impairment. We sought (1) to examine the combined effects of HTN and CD33, an immune‐related and robust cognitive impairment susceptibility locus, with cognitive function in older adults and further (2) to explore the underlying mechanisms.

**Method:**

This population‐based study included 4444 dementia‐free participants (age ≥65 years) in MIND‐China; of these, data on gray matter volume were available in 1047 persons and cerebral blood flow (CBF) in 85 persons. We used general linear regression models to examine the associations of CD33 rs3865444 and HTN with cognition, brain atrophy, and CBF, and the mediation models to explore associations among them.

**Result:**

Among participants carrying CD33 rs3865444 CC genotype, HTN history, high pulse pressure, and late‐life HTN were significantly associated with low cognitive performance, HTN history as well as midlife HTN was correlated with small volume of gray matter. Notably, rs3865444 CC genotype significantly moderated the mediation effect of gray matter volume on the association between HTN and global cognition (mediation effect: 9.33%). Furthermore, HTN was correlated with low CBF in individuals carrying rs3865444 CC. Similar results were obtained among individuals free of mild cognitive impairment.

**Conclusion:**

The current study reveals the synergistic associations of immune‐related CD33 rs3865444 and HTN with low cognitive function and brain atrophy in dementia‐free older adults. These joint effects of HTN and CD33 on cognition may be related to the disruption of CBF and blood brain barrier function.

